# Bromodomain inhibition shows antitumoral activity in mice and human luminal breast cancer

**DOI:** 10.18632/oncotarget.18255

**Published:** 2017-05-29

**Authors:** Montserrat Pérez-Salvia, Laia Simó-Riudalbas, Pere Llinàs-Arias, Laura Roa, Fernando Setien, Marta Soler, Manuel Castro de Moura, James E. Bradner, Eva Gonzalez-Suarez, Catia Moutinho, Manel Esteller

**Affiliations:** ^1^ Cancer Epigenetics and Biology Program (PEBC), Bellvitge Biomedical Research Institute (IDIBELL), Barcelona, Catalonia, Spain; ^2^ Novartis Institutes for Biomedical Research, Cambridge, MA, USA; ^3^ Physiological Sciences Department, School of Medicine and Health Sciences, University of Barcelona (UB), Barcelona, Catalonia, Spain; ^4^ Institució Catalana de Recerca i Estudis Avançats (ICREA), Barcelona, Catalonia, Spain

**Keywords:** luminal breast cancer, bromodomain inhibitor, C-MYC, JQ1, mice model

## Abstract

BET bromodomain inhibitors, which have an antitumoral effect against various solid cancer tumor types, have not been studied in detail in luminal breast cancer, despite the prevalence of this subtype of mammary malignancy. Here we demonstrate that the BET bromodomain inhibitor JQ1 exerts growth-inhibitory activity in human luminal breast cancer cell lines associated with a depletion of the C-MYC oncogene, but does not alter the expression levels of the BRD4 bromodomain protein. Interestingly, expression microarray analyses indicate that, upon JQ1 administration, the antitumoral phenotype also involves downregulation of relevant breast cancer oncogenes such as the Breast Carcinoma-Amplified Sequence 1 (BCAS1) and the PDZ Domain-Containing 1 (PDZK1). We have also applied these *in vitro* findings in an *in vivo* model by studying a transgenic mouse model representing the luminal B subtype of breast cancer, the MMTV-PyMT, in which the mouse mammary tumor virus promoter is used to drive the expression of the polyoma virus middle T-antigen to the mammary gland. We have observed that the use of the BET bromodomain inhibitor for the treatment of established breast neoplasms developed in the MMTV-PyMT model shows antitumor potential. Most importantly, if JQ1 is given before the expected time of tumor detection in the MMTV-PyMT mice, it retards the onset of the disease and increases the survival of these animals. Thus, our findings indicate that the use of bromodomain inhibitors is of great potential in the treatment of luminal breast cancer and merits further investigation.

## INTRODUCTION

Breast cancer is a leading cause of cancer death in women, estimated to account for more than 450,000 deaths worldwide every year [1]. Despite the improved early detection of the disease and the new therapies, the major health concern associated with breast cancer persists probably due to several factors, among them the biological heterogeneity of the pathology. Classical clinical and pathological markers, such as the status of the estrogen and progesterone receptors and human epidermal growth factor 2 (HER2) gene amplification, are useful for classifying patients according to prognosis and adequate treatments, but it has been the emergence of genomic technologies, such as global expression profiling, that has allowed an intrinsic molecular classification [2–4]. In this regard, five distinct intrinsic molecular subtypes are recognized: luminal A, luminal B, HER-2 enriched, basal-like and claudin-low, together with a normal breast-like group [2–4]. These subgroups relate to the clinically used immunohistochemical classification and, for example, luminal A is estrogen receptor- and/or progesterone receptor-positive but with a low Ki-67 index, whereas luminal B is estrogen receptor- and/or progesterone receptor-positive and high Ki-67 index. Overall, the majority of breast tumors are positive for the hormone receptors and, thus, amenable to endocrine therapies. However, in the natural history of the disease, progression is associated with the acquisition of resistance to the endocrine treatment, limiting the efficacy of these pharmacological compounds. In addition, the survival curves of luminal B subtype cross those of basal-like disease at ten years [4]. Thus, given the high frequency of luminal breast cancer, the generation of endocrine therapy-associated resistances and the poor outcome of the luminal B subtype, the development of new drugs for these patients is essential.

One interesting avenue to explore is the targeting of the epigenome of breast cancer cells. In this regard, DNA demethylating agents and histone deacetylase inhibitors have been clinically approved for certain subtypes of leukemias and lymphomas [5]. New promising agents are compounds that can block the “reading” of the acetylated histone marks, preventing the active transcription of growth-promoting genes. This is the case of the BET bromodomain inhibitors that remove BET bromodomain proteins from their chromatin targets by competing with acetylated histone residues [6]. The patterns of histone acetylation shifts in human tumors [7] and recent data have shown that the BET bromodomain inhibitor inhibits the growth of triple-negative breast cancer cell lines and xenografts [8–15]. Thus, we sought to determine whether BET bromodomain inhibitors were also effective not only in luminal breast cancer cell lines, but also in a mouse model of luminal B breast cancer (MMTV-PyMT) [16]. Furthermore, we also characterized gene targets involved in the antiproliferative effect mediated by the BET bromodomain inhibitor JQ1 [17].

## RESULTS

### The bromodomain inhibitor JQ1 decreases cell viability of human luminal breast cancer cell lines in association with downregulation of C-MYC and mammary oncogenic proteins

**Figure 1:**
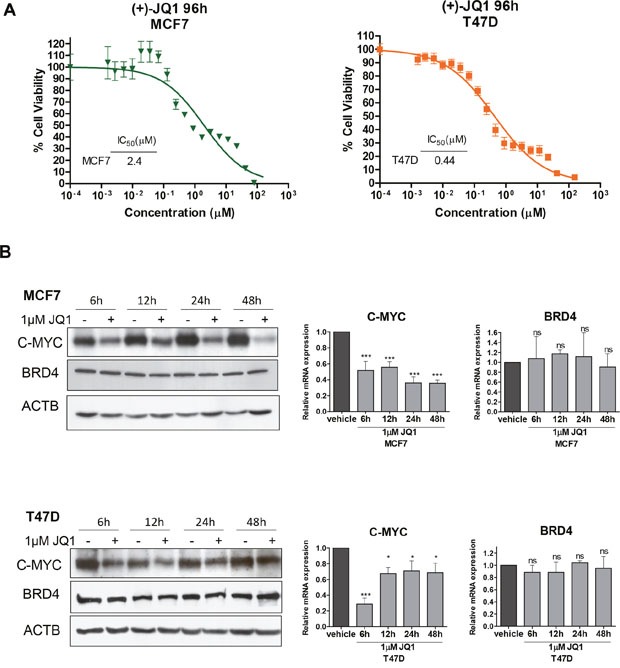
JQ1 treatment of human luminal breast cancer cell lines reduces cell viability and downregulates C-MYC **(A)** Effect of JQ1 on cell viability determined by the MTT assay in MCF7 and T47D cells. The corresponding half-maximal inhibitory concentration (IC50) values are shown for each cell line. **(B)** Downregulation of C-MYC upon JQ1 treatment (1 μM) at 6, 12, 24 and 48 h in breast cancer cell lines, as determined by Western blot (left) and qRT-PCR (right). BRD4 expression levels do not change. *P<0.05; ***P<0.001; ns: non-significant.

To study the cellular impact of JQ1 as a candidate growth inhibitor for luminal breast cancer, we chose the initial biological model of two human cancer cell lines with a well characterized luminal phenotype, MCF7 and T47D [18, 19]. Using the 3-(4,5-dimethyl-2-thiazolyl)-2,5-diphenyl-2H-tetrazolium bromide (MTT) assay to measure cell viability in the chosen cancer cell lines, we observed that JQ1 inhibited cancer cell growth dose-dependently (Figure 1A). Interestingly, IC50 sensitivity to this first-in-class BET bromodomain inhibitor compound is in the same range as that of other antitumoral drugs that target histone modifications, such as histone deacetylases [20] and histone kinases [21, 22]. The next step was to show that the growth inhibition-mediated effect of the JQ1 compound occurred in the context of the induced downregulation of C-MYC, as has been described in other models [23, 24]. Western blot and quantitative reverse transcription-polymerase chain reaction (qRT-PCR) demonstrated that JQ1 diminished C-MYC expression levels (Figure 1B) in both luminal breast cancer cell lines. As expected [17, 23, 24], the use of JQ1 did not modify the expression levels of the bromodomain protein BRD4, as also shown by western blot and qRT-PCR (Figure 1B).

**Figure 2:**
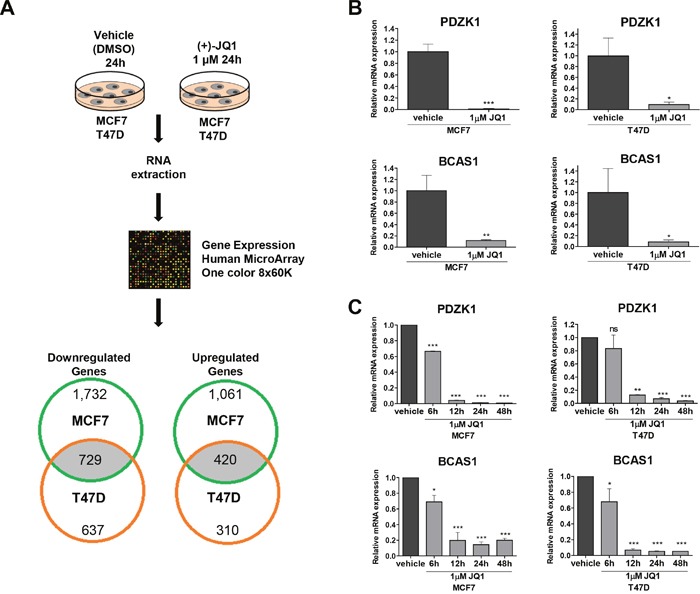
Expression microarray analyses identify PDZK1 and BCAS1 as breast cancer oncoproteins downregulated by JQ1 treatment in human luminal breast cancer cell lines **(A)** Flowchart of the expression microarray experiment. **(B)** qRT-PCR validation of the microarray results for PDZK1 and BCAS1. **(C)** Downregulation of PDZK1 and BCAS1, determined by qRT-PCR, upon JQ1 use at different times. *P<0.05; **P<0.01; ***P<0.001.

We next sought to identify other gene targets that, in addition to the well characterized depletion of the C-MYC oncogene, could also explain the observed growth inhibitory effect of the use of the BET bromodomain inhibitor. We performed an expression microarray experiment in MCF7 and T47D cells treated with a vehicle compared with those in which we used the JQ1 compound (Figure 2A). The expression microarray data have been deposited in the Gene Expression Omnibus (GEO) repository under Accession Number GSE95287. Of the set of 36,712 unique genes included in the microarray, 1,149 (3.1%) significantly changed in a shared manner in both cell lines; 420 (36.6%) of them were upregulated and 729 (63.4%) were downregulated. The latter set constitutes the putative direct substrates of JQ1 that act by removing the BET bromodomain proteins from their regulatory regions through competition with acetylated histone residues that are usually associated with active transcription [6]. Gene functional annotation analysis for these transcripts was performed by computing gene overlapping with GSEA KEGG and GO signature collections. Among the 729 genes downregulated upon JQ1 use, we observed an overrepresentation of KEGG pathways and GO biological process terms related with pathways in cancer and regulation of cell proliferation, respectively (False Discovery Rate q-value <0.05) (Supplementary Figure 1 and Supplementary Table 1). Among the top candidate genes commonly downregulated, our attention was particularly drawn by the presence of the transcripts for the PDZ Domain-Containing 1 (PDZK1) and the Breast Carcinoma-Amplified Sequence 1 (BCAS1) genes, which encode two oncoproteins that have been linked to mammary tumorigenesis [25–28]. PDZK1 promotes estrogen-mediated growth of breast cancer cells [25, 26], whereas BCAS1 undergoes gene amplification-associated overexpression in breast cancer [27] and has been implicated in breast cancer progression [28]. The downregulation of these transcripts upon JQ1 administration was validated by qRT-PCR in the same RNA extracts as used for microarray hybridization (Figure 2B) and when the experiment was repeated in a new batch of cells treated for different times (Figure 2C). We also assessed to what extent the effects of JQ1 are mediated by its silencing of C-MYC compared to PDZK1 and BCAS1. Upon efficient short hairpin RNA (shRNA) mediated dowregulation of MYC, PDZK1 or BCAS1 in MCF7 and T47D (Supplementary Figure 2A), we observed a similar significant decrease in the cell viability determined by the MTT assay (Supplementary Figure 2B and 2C). These data suggest that all three proteins exert a similar role as growth promoting factors in these luminal breast cancer cell lines. Importantly, the growth inhibition mediated by the use of JQ1 is higher than the one observed for the depletion of each factor (Supplementary Figure 2B and 2C). Thus, the observed growth-inhibitory effects of bromodomain inhibition in the luminal cells studied can be explained not only by the diminished expression of C-MYC, but also by a global reduction in the levels of transforming genes such as those exemplified by the PDZK1 and BCAS1 breast cancer oncogenes.

### Bromodomain inhibition shows antitumoral activity and retards the onset of luminal breast tumors in the MMTV-PyMT luminal breast cancer mouse model

**Figure 3:**
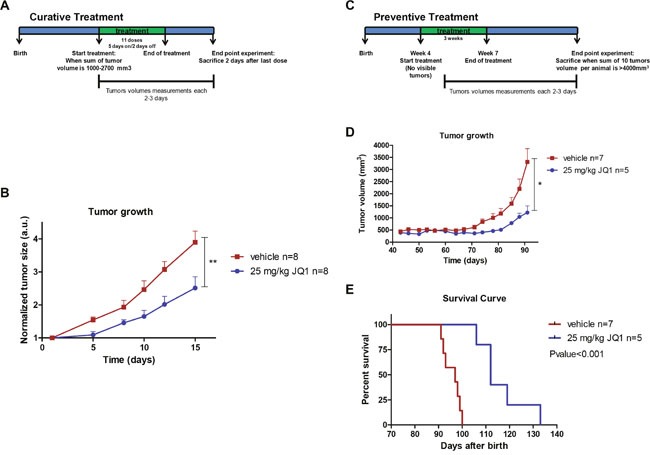
JQ1 treatment inhibits growth and prevents formation of luminal breast tumors in the MMTV-PyMT mouse model **(A)** Design of the “curative treatment experiment”. **(B)** Tumor volume monitored over time in vehicle- and JQ1-treated MMTV-PyMT mice. **P<0.01. **(C)** Design of the “prevention treatment experiment”. **(D)** Tumor volume monitored over time in vehicle- and JQ1-treated MMTV-PyMT mice. *P<0.05. **(E)** Kaplan-Meier survival curves for vehicle- and JQ1-treated MMTV-PyMT mice. Statistical differences tested with Log Rank (Mantel-Cox) test.

We next transferred our experiments from the *in vitro* and cell line assays described above to the *in vivo* setting in a mouse model of luminal breast cancer. The antitumoral activity of JQ1 was evaluated using MMTV-PyMT transgenic mice that spontaneously develop multifocal luminal B breast tumors [16]. To assess the efficacy of JQ1 at inhibiting the growth of established tumors, we started the treatment when the total tumor volumes of each animal reached ~ 1,000-2,700 mm^3^ (Figure 3A). We randomly selected eight MMTV-PyMT mice as the control group treated with vehicle and another eight for JQ1 treatment (25 mg/kg). Tumor volume was monitored every 2-3 days. The lack of toxicity of the drug was found under the described conditions. The use of the BET bromodomain inhibitor was significantly associated with the development of smaller breast tumors than those that occurred in the control group (Figure 3B).

Finally, once we had established the efficacy of JQ1 at inhibiting the growth of established luminal breast tumors, we considered whether the drug not only had a therapeutic, but also a preventive effect. The impact of BET bromodomain inhibition on preventing spontaneous mammary tumors that naturally arise in MMTV-PyMT mice was evaluated as described in Figure 3C. Briefly, the MMTV-PyMT mice were randomly divided into a treatment group (25 mg/kg, n=5) and a vehicle group (n=7). JQ1 was administered when mice were 4 weeks old and when no palpable or visible tumors existed. Tumor volume was monitored every 2-3 days. We observed that those MMTV-PyMT mice receiving the BET bromodomain inhibitor experienced later onset of breast cancer and developed significantly smaller tumors (Figure 3D). Most notably, the treatment with JQ1 increased the overall survival of the MMTV-PyMT mice in comparison to those that received the mock treatment (Figure 3E). These results suggest that bromodomain inhibitors may exert a protective anti-tumorigenic effect against tumorigenesis, and that it would be worthwhile exploring the benefits of using them in the context of individuals with a high-risk of developing breast cancer and other malignancies.

## DISCUSSION

Herein, we have analyzed the effect of the BET bromodomain inhibitor JQ1 in the context of luminal breast cancer in mouse models and human cells. Our results highlight how using the epigenetic drug yields remarkable antitumoral effects against luminal breast tumors in association with the downregulation of its known target C-MYC. These findings represent the first demonstration *in vivo* of the antiproliferative characteristics of this small molecule for this particular mammary cancer subtype. Importantly, the impact of JQ1 on the transcriptional landscape of the treated breast cancer cells extends beyond the depletion of C-MYC to affect hundreds of other genes. Among the candidates that can also mediate the growth inhibitory action of the compound, we have further characterized the JQ1-associated downregulation of two important breast cancer oncogenes, BCAS1 and PDZK1. Our findings suggest that the anticancer effect observed for the BET bromodomain inhibitor involves many cellular and signaling pathways and that the target genes can have tumor-type-specific patterns.

Our study also provides at least another interesting indication of the significant role of bromodomain proteins in tumorigenesis. We show that the use of the BET bromodomain inhibitor JQ1 prevents the development of breast cancer in mice. Our results demonstrate that the administration of JQ1 in the MMTV-PyMT significantly delayed the development of breast tumors and increased overall survival. Notably, the treatment of the mice with the epigenetic drug did not result in any evident adverse developmental consequences in these animals. These results, in addition to identifying a key role for bromodomain proteins in breast carcinogenesis, are encouraging as proof-of-concept that these types of compound may be useful in cancer chemoprevention strategies. In this regard, it would be worth pre-clinically testing the efficacy of BET bromodomain inhibitors in diminishing the onset of disease in women at high-risk of developing breast cancer, such as those that are carriers of germline mutations in the tumor suppressor and DNA repair genes BRCA1 and BRCA2. Interestingly, recent findings indicate that the presence of BRCA1 mutations is associated with augmented proliferation of luminal progenitor cells [29–32] and, thus, JQ1 could be used in pre-neoplastic tissue to block these hyperactive cells in their course towards full cancer development.

Finally, it is relevant to mention that although JQ1 was the first-in-class BET bromodomain inhibitor, other drugs have been developed with similar or identical targets [6], such as I-BET151 [33], RVX-208 [34] and OTX015 [15, 35], that can work in the breast cancer models studied and that could be tested. However, all these drugs have common features that interfere with the binding of bromodomain protein to their targets in normal and cancer cells, so far with little specificity. It is in this context that the necessity might emerge for a more personalized cancer treatment using BET bromodomain inhibitors based on the genomic alterations observed in individual patients. In this regard, the presence of point mutations, gene copy number alterations and translocations involving histone acetyltransferases, histone deacetylases and the bromodomain proteins themselves [36–38] could be important biomarkers for predicting the true therapeutic potential of these drugs.

## MATERIALS AND METHODS

### Cell lines

The human luminal breast cancer cell lines MCF-7 and T47D used in this study were purchased from the American Type Culture Collection (ATCC). MCF-7 and T47D were cultured in DMEM and RPMI, respectively. Both mediums were supplemented with 10% fetal bovine serum and the cells were grown at 37°C and 5% CO_2_.

### Dose-response assays

For dose-response assays, 3000 cells were seeded in 96-well plates. The optimal number of cells for each experiment was determined to ensure that each one was in growth phase at the assay endpoint. After overnight incubation, experimental medium containing increasing concentrations of JQ1 was added into each well. Cell viability assay was determined at 96 h after treatment, by the 3-(4,5-dimethyl-2-thiazolyl)-2,5-diphenyl-2H-tetrazolium bromide (MTT) assay. Briefly, MTT reagent was added and incubated for 3 h, after which the cells were lysed for 16 h with MTT lysis buffer (50% N-N dimethylformamide, 20% sodium dodecyl sulfate, 2.5% glacial acetic acid, 2.1% 1N HCl, at pH 4.7). Plates were measured at 560 nm using a spectrophotometer.

### RNA isolation and quantitative PCR

Total RNA was extracted using a Maxwell® RSC simply RNA Cell Kit (Promega). Real-time PCR reactions were performed following the methods for use of SYBR Green (Applied Biosystems). GAPDH was used as an endogenous control to enable normalization. Specific primers are detailed in Supplementary Table 2.

### Immunoblotting assays

Total protein from cells was extracted with Laemmli sample buffer (62.5 mM Tris-HCl pH 6.8, 25% glycerol, 2% SDS, 0.01% bromophenol blue, 5% β-mercaptoethanol). Specific antibodies against target proteins are detailed in Supplementary Table 2.

### Gene expression microarray analysis

For expression array analysis, twelve RNA samples were extracted using a Maxwell® RSC simply RNA Cell Kit (Promega) and sent to the CRG Genomics Unit (Barcelona). RNA was extracted from three independent biological replicates treated with JQ1 (1 μM 24h) and three independent biological replicates treated with vehicle (DMSO). Expression data from the Agilent Gene Expression one-color chip human 8×60K microarrays were analyzed with the Bioconductor limma library v3.28 in the R v3.3.0 statistical environment. Briefly, the extracted intensities were background-corrected by applying the normexp calculation. The background-corrected log_2_-transformed values were quantile-normalized to make data from all arrays comparable. After filtering out control and low-level expression probes, we applied empirical Bayes statistics within the limma package for two class comparisons in order to calculate the difference in expression between conditions. Transcripts with significant differences (absolute logFC > 1 and adjusted p < 0.05) were considered for further analysis. The gene functional annotation analysis was performed by computing gene overlapping with GSEA KEGG and GO signature collections. We used a hypergeometric test to assess the overrepresentation of specific functions in the gene set tested. The associated hypergeometric p-value was corrected for multiple hypotheses testing according to Benjamini and Hochberg. Finally, we selected the 10 most significant over-represented terms with a False Discovery Rate q-value below 0.05.

### Short hairpin interference

Two different sequence gene specific hairpin RNA molecules (shRNAs) for C-MYC, PDZK1, or BCAS1 mRNA were designed and transduced into MCF7 and T47D breast cancer cell lines. shRNA against the MSS2 yeast protein (not present in mammals) was used as scrambled (control). shRNAs and scramble sequences can be found in Supplementary Table 2. All shRNA molecules were ligated into pLVX-shRNA2-ZsGreen plasmid from Clontech, using BamHI and EcoRI restriction enzymes. Each shRNA-encoding plasmid (10 μg) was mixed with 7.5 μg of ps-PAX2 and 2.5 μg of PMD2.G plasmid in 1 ml JetPRIME buffer and 50 μl of JetPRIME. Upon 10 min of RT incubation, the transfection mix was added dropwise on a 10 cm culture plate containing HEK293-TLV lentiviral packaging cells at 80% confluence. After 72 h, medium with high-titer lentiviral particles was 0.45 μm filtered. MCF7 and T47D target cells were cultured in virus containing medium for 24 h. As transduction efficiencies were higher than 95%, and in order to avoid the cloning bias-effect, we chose working with a pool of cells with high expression of shRNA constructs. Cell proliferation was determined by the MTT assay. A total of 1000 cells of MCF7 or 2000 cells of T47D were plated onto 96-well plates in the corresponding medium with vehicle or with 1μM JQ1. MTT was added on 8 consecutive days at a final concentration of 5 mg/mL and further procedure was done as described previously.

### Mouse model

MMTV-PyMT^+^ male mice (FVB/Nj strain) were kindly provided by Dr. Gonzalez-Suarez (IDIBELL, Barcelona, Spain). Transgenic females were obtained by breeding FVB/Nj females with PyMT^+^ transgenic males. All mouse experiments were approved by the IDIBELL Animal Care and Use Committee and performed in accordance with the guidelines of The International Guiding Principles for Biomedical Research Involving Animals, developed by the Council for International Organizations of Medical Sciences (CIOMS).

### Curative *in vivo* treatment

When the total tumor volume of each animal reached ~ 1,000-2,700 mm^3^, PyMT^+^ female mice were randomized into a JQ1 treatment group (25 mg/kg, n=8) and a vehicle (control) group (n=8). JQ1 or vehicle (1:10 DMSO:10% hydroxypropyl β cyclodextrin) was administered daily intraperitoneally for 11 doses on a 5-days-on/2-days-off dosing schedule. Tumor growth was monitored every 2-3 days by measuring tumor width (*W*) and length (*L*). Tumor volume, *V*, was then estimated from the formula *V*=π/6x (*L* x *W*^2^) and reported as the sum of all the tumor volumes for each animal and the mean and SEM of each mouse group. Two days after completion of the treatment, mice were euthanized.

### Preventive *in vivo* treatment

PyMT^+^ female mice were randomly divided into a JQ1 treatment group (25 mg/kg, n=5) and a vehicle (control) group (n=7). When mice were 4 weeks old and no palpable or visible tumors were present, JQ1 or vehicle (1:10 DMSO:10% hydroxypropyl β cyclodextrin) were administered intraperitoneally daily for 3 weeks on a 5-days-on/2-days-off dosing schedule. Tumor growth was monitored every 2-3 days by measuring tumor width (*W*) and length (*L*). Tumor volume, *V*, was then estimated from the formula *V*=π/6x(*L* x *W*^2^). Mice were euthanized when the total 10 tumor volume per animal was greater than 4,000 mm^3^.

### Statistical analysis

Real Time Quantitative PCR results were statistically analyzed with a Two samples T test, in the case where JQ1 treated and not treated samples were compared an din the case of shRNA depletion; with a Tukey Multiple comparasions of mean test, in time course experiments cases. Concerning tumor and cell growth experiments we used an AUC Vardi test with 1000 permutations. Kaplan-Meier survival curves statistical differences were tested with Log Rank (Mantel-Cox) test. P values less than 0.05 were considered significant (*P>0.05; **P>0.005; ***P>0.001; n.s = no significance).

## SUPPLEMENTARY MATERIALS FIGURES AND TABLES


