# Supplementary material for: Bromodomain inhibition shows antitumoral activity in mice and human luminal breast cancer
Source: Oncotarget. 2017 May 29;8(31):51621–9. doi: 10.18632/oncotarget.18255 (PMC5584274; doi:10.18632/oncotarget.18255)
Supplement: Supplementary file 1 [file oncotarget-08-51621-s001.pdf]

Bromodomain inhibition shows antitumoral activity in mice and human luminal breast cancer

SUPPLEMENTARY MATERIALS

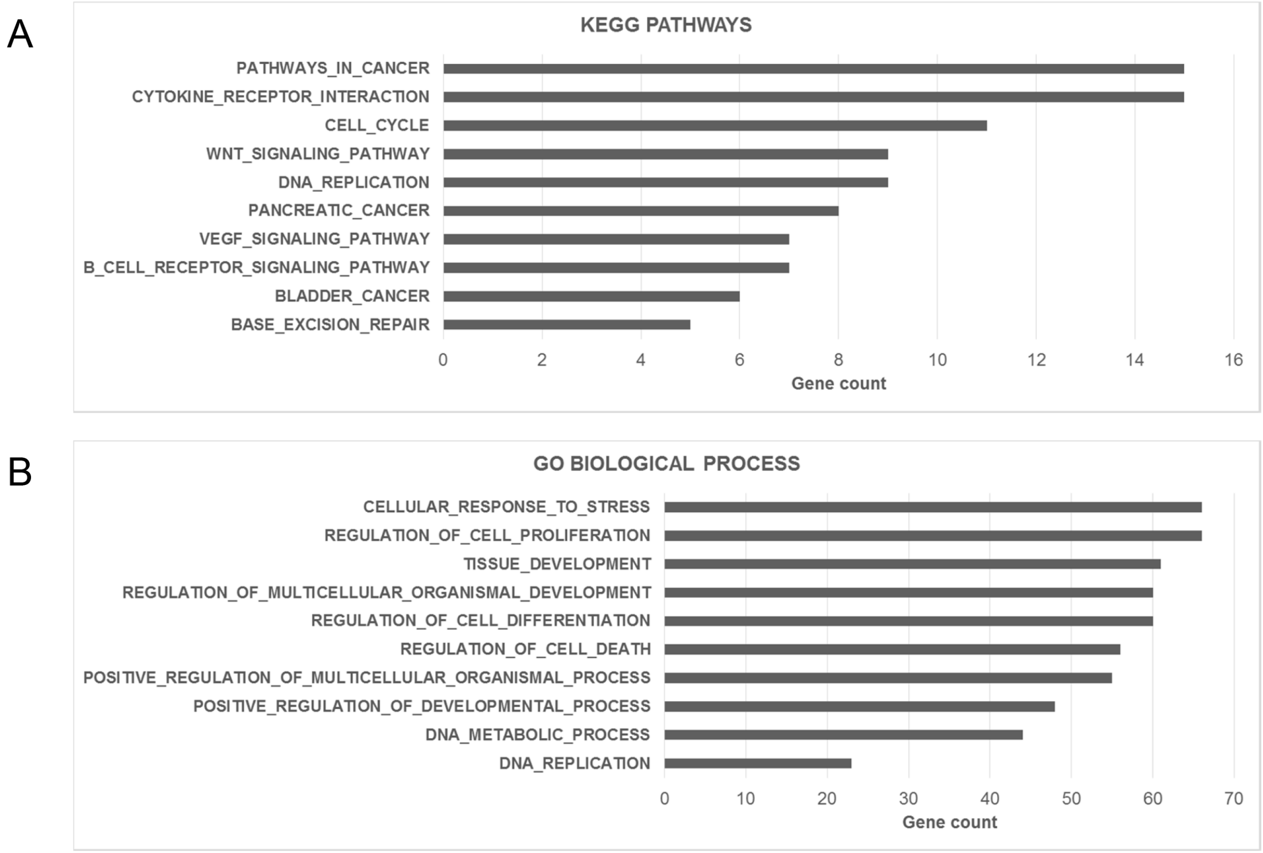

Supplementary Figure 1: Gene functional annotation analyses in both MCF7 and T47D breast cancer cell lines for the downregulated gene set upon JQ1 use. **(A)** Overrepresented KEGG pathways, **(B)** GO biological process terms.

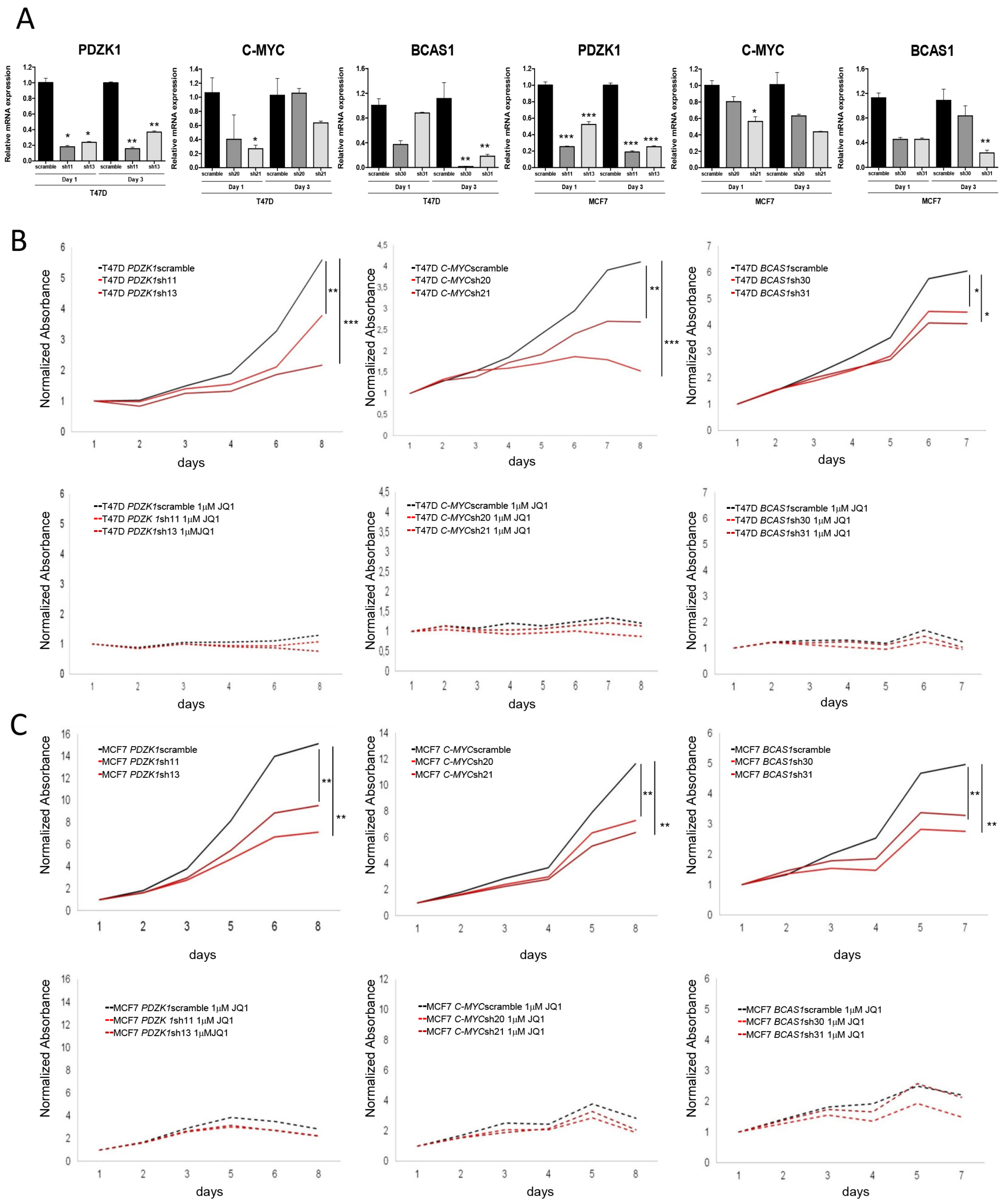

Supplementary Figure 2: Short harpin RNA (shRNA) mediated dowregulation of C-MYC, PDZK1 or BCAS1 in luminal breast cancer cell lines. (A) Quantitative RT-PCR analyses to show the effficient depletion of the target genes upon shRNA use. Effect on cell growth determined by the MTT assay of the shRNA-mediated depletion of C-MYC, PDZK1 or BCAS1 in T47D **(B)** and MCF7 **(C)** cells. Scramble shRNA and two target specific shRNAs for each gene are shown. The use of JQ1 treatment in these shRNAs transduced cells is also represented. *P<0.05; **P<0.01; ***P<0.001.

Supplementary Table 1: KEGG and GO analyses

Gene set name	# Genes in gene set (K)	# Genes in overlap (k)	k/K	p-value	FDR q-value
KEGG_DNA_REPLICATION	36	9	0.25	3.13E-10	5.82E-08
KEGG_CELL_CYCLE	128	11	0.0859	4.19E-07	3.90E-05
KEGG_CYTOKINE_RECEPTOR_INTERACTION	267	15	0.0562	9.09E-07	5.63E-05
KEGG_PANCREATIC_CANCER	70	8	0.1143	1.87E-06	8.68E-05
KEGG_BLADDER_CANCER	42	6	0.1429	9.98E-06	3.44E-04
KEGG_PATHWAYS_IN_CANCER	328	15	0.0457	1.11E-05	3.44E-04
KEGG_B_CELL_RECEPTOR_SIGNALING_PATHWAY	75	7	0.0933	3.18E-05	8.05E-04
KEGG_VEGF_SIGNALING_PATHWAY	76	7	0.0921	3.46E-05	8.05E-04
KEGG_BASE_EXCISION_REPAIR	35	5	0.1429	5.62E-05	1.16E-03
KEGG_WNT_SIGNALING_PATHWAY	151	9	0.0596	8.77E-05	1.62E-03
GO_REGULATION_OF_CELL_PROLIFERATION	1496	66	0.0441	6.90E-20	3.06E-16
GO_CELLULAR_RESPONSE_TO_STRESS	1565	66	0.0422	6.93E-19	1.54E-15
GO_DNA_METABOLIC_PROCESS	758	44	0.058	8.05E-18	1.19E-14
GO_TISSUE_DEVELOPMENT	1518	61	0.0402	1.55E-16	1.72E-13
GO_REGULATION_OF_CELL_DIFFERENTIATION	1492	60	0.0402	2.71E-16	2.40E-13
GO_DNA_REPLICATION	208	23	0.1106	9.84E-16	7.27E-13
GO_POSITIVE_REGULATION_OF_MULTICELLULAR_ORGANISMAL_PROCESS	1395	55	0.0394	1.17E-14	7.43E-12
GO_REGULATION_OF_CELL_DEATH	1472	56	0.038	2.90E-14	1.61E-11
GO_REGULATION_OF_MULTICELLULAR_ORGANISMAL_DEVELOPMENT	1672	60	0.0359	4.13E-14	2.04E-11
GO_POSITIVE_REGULATION_OF_DEVELOPMENTAL_PROCESS	1142	48	0.042	6.27E-14	2.78E-11

Top, Overrepresented KEGG pathways corresponding to the significant downregulated genes in MCF7 and T47D cell lines. The table shows the genes overlapping each gene set and the associated hypergeometric test's p-value. *Below*, Overrepresented GO biological process terms corresponding to the significant downregulated genes in MCF7 and T47D cell lines. The table shows the genes overlapping each gene set and the associated hypergeometric test's p-value.

Supplementary Table 2: Primers for qRT-PCR, antibodies used in the western blot and shRNAs sequences

**Primers for qRT-PCR**				
BRD4_Hs_qPCR_F	CAACAAGCCTGGAGATGACA			
BRD4_Hs_qPCR_R	GGAGGAGTCGATGCTTGAGT			
MYC_Hs_qPCR_F	CCGCTTCTCTGAAAGGCTCT			
MYC_Hs_qPCR_R	AAGCTAACGTTGAGGGGCAT			
PDZK1_Hs_qPCR_F	AAACTCTGCAGGCTGGCTAA			
PDZK1_Hs_qPCR_R	TCCACCACCTTCTCATAGGG			
BCAS1_Hs_qPCR_F	CCAGAAGGACTGGAGACTGC			
BCAS1_Hs_qPCR_R	CTTGGGTCTCCTGGGATGTA			
GAPDH_Hs_qPCR_F	GAAGGTGAAGGTCGGAGTCA			
GAPDH_Hs_qPCR_R	TGGACTTCACGACGTACTCA			
**Antibodies name**	**Reference**	**Company**	**Application**	**Dilution**
Anti-BRD4	ab128874	Abcam	WB	1:1000
C-MYC (D84C12)	5605	Cell signaling	WB	1:1000
Actin-beta HRP	A3854	Sigma	WB	1:20000
**Lentiviral shRNAs**				
Scramble_F	gatccGCGCAGAACAAATTCGTCCATTCAAGAGATGGACGAATTTGTTCTGCGTTTTTTACGCGTg			
Scramble_R	aattcACGCGTAAAAAACGCAGAACAAATTCGTCCATCTCTTGAATGGACGAATTTGTTCTGCGCg			
PDZK1_sh11_F	gatccGCCTTCAAGATGGAGACAGATTCAAGAGATCTGTCTCCATCTTGAAGGTTTTTTACGCGTg			
PDZK1_sh11_R	aattcACGCGTAAAAAACCTTCAAGATGGAGACAGATCTCTTGAATCTGTCTCCATCTTGAAGGCg			
PDZK1_sh13_F	gatccGGTGGACTTGAAAGAGTTGTTCAAGAGACAACTCTTTCAAGTCCACCTTTTTTACGCGTg			
PDZK1_sh13_R	aattcACGCGTAAAAAAGGTGGACTTGAAAGAGTTGTCTCTTGAACAACTCTTTCAAGTCCACCg			
MYC_sh20_F	gatccGCACGAAACTTTGCCCATAGTTCAAGAGACTATGGGCAAAGTTTCGTGTTTTTTACGCGTg			
MYC_sh20_R	aattcACGCGTAAAAAACACGAAACTTTGCCCATAGTCTCTTGAACTATGGGCAAAGTTTCGTGCg			
MYC_sh21_F	gatccGCTTCACCAACAGGAACTATTCAAGAGATAGTTCCTGTTGGTGAAGCTTTTTTACGCGTg			
MYC_sh21_R	aattcACGCGTAAAAAAGCTTCACCAACAGGAACTATCTCTTGAATAGTTCCTGTTGGTGAAGCg			
BCAS1_sh30_F	gatccACCAGAAGCAGAGACTTACTTCAAGAGAGTAAGTCTCTGCTTCTGGTTTTTTTACGCGTg			
BCAS1_sh30_R	aattcACGCGTAAAAAAACCAGAAGCAGAGACTTACTCTCTTGAAGTAAGTCTCTGCTTCTGGTg			
BCAS1_sh31_F	gatccGCACACAGTTCAGCACTTAGTTCAAGAGACTAAGTGCTGAACTGTGTGTTTTTTACGCGTg			
BCAS1_sh31_R	aattcACGCGTAAAAAACACACAGTTCAGCACTTAGTCTCTTGAACTAAGTGCTGAACTGTGTGCg			
